# Giant Retroperitoneal Liposarcoma: A Rare Surgical Occurrence

**DOI:** 10.7759/cureus.76192

**Published:** 2024-12-22

**Authors:** Dakshayani S Nirhale, Vidita Modi, Aditya A Kulkarni

**Affiliations:** 1 General Surgery, Dr. D Y Patil Medical College, Hospital and Research Centre, Dr. D Y Patil Vidyapeeth (Deemed to be University), Pune, IND

**Keywords:** complete resection, giant, liposarcoma, recurrence, retroperitoneal

## Abstract

Retroperitoneal liposarcoma is a rare malignancy that arises from adipocytes and can expand significantly before manifesting clinical symptoms. Instances of giant retroperitoneal liposarcoma, defined as tumors larger than 30 cm, are extremely rare, with fewer than 20 reported cases. This case report presents a 68-year-old patient with a significant abdominal mass, ultimately identified and treated as a well-differentiated retroperitoneal liposarcoma. The case involves the rarity of the disease, making its diagnosis and management difficult due to the presence of large tumors. Additionally, postoperative care for such cases is also quite challenging.

## Introduction

Soft tissue sarcomas are extremely rare, making up less than 1% of all adult cancers. Liposarcomas can form in any location where fatty tissue is present, with retroperitoneal liposarcomas accounting for 12% to 40% of all liposarcoma cases [1], and an incidence rate of approximately 0.5 per 100,000 individuals [2]. Due to the expansive nature of the retroperitoneal space, these tumors can grow significantly before detection, often involving adjacent organs and vessels and leaving patients asymptomatic for extended periods [3]. Tumors weighing over 20 kg are classified as "giant liposarcomas" and are exceptionally rare [4]. Given their rarity and the complexity associated with their size, treatment experience for such tumors is limited.

## Case presentation

**Figure 1:**
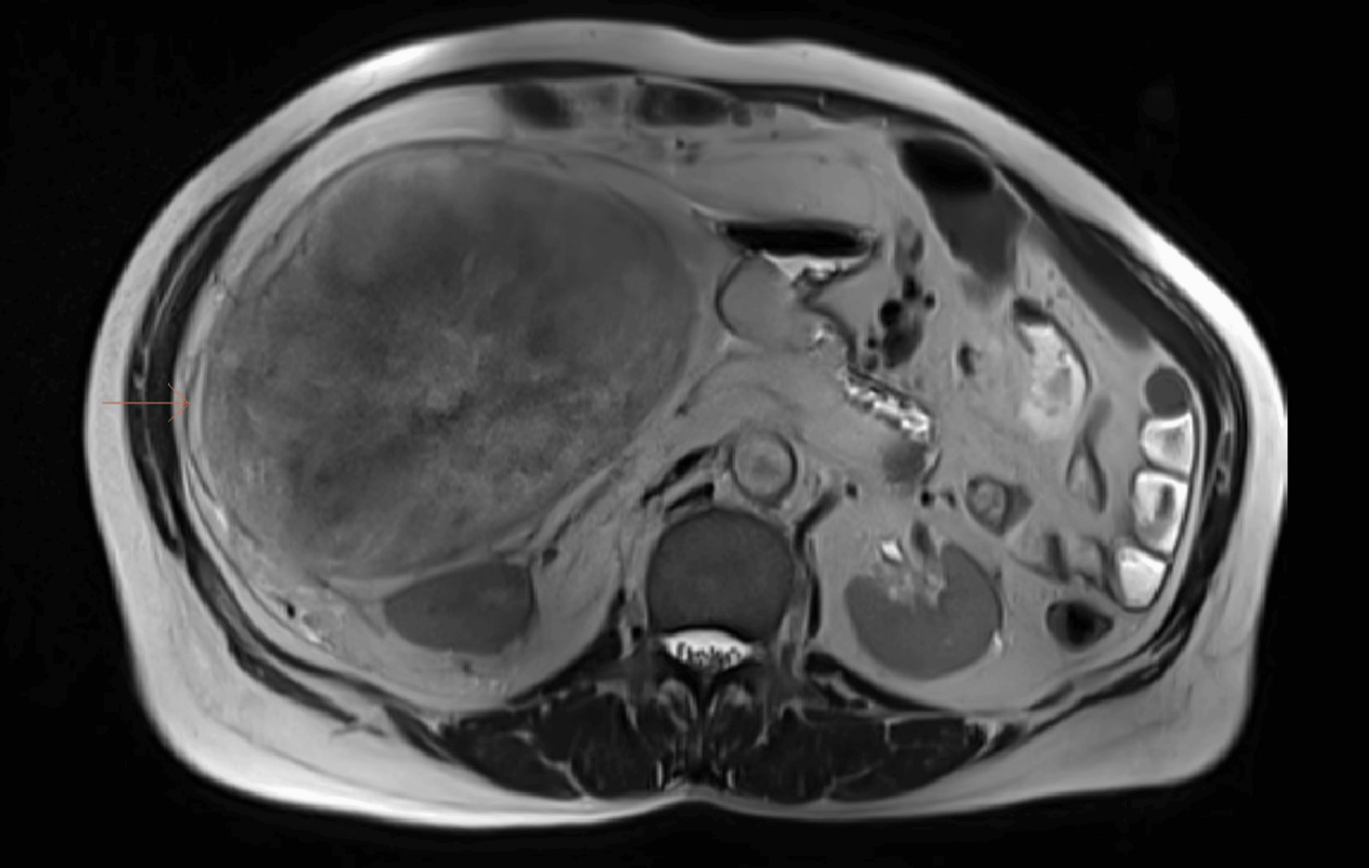
MRI T2WI axial section showing retroperitoneal mass. T2WI, T2-weighted imaging.

**Figure 2:**
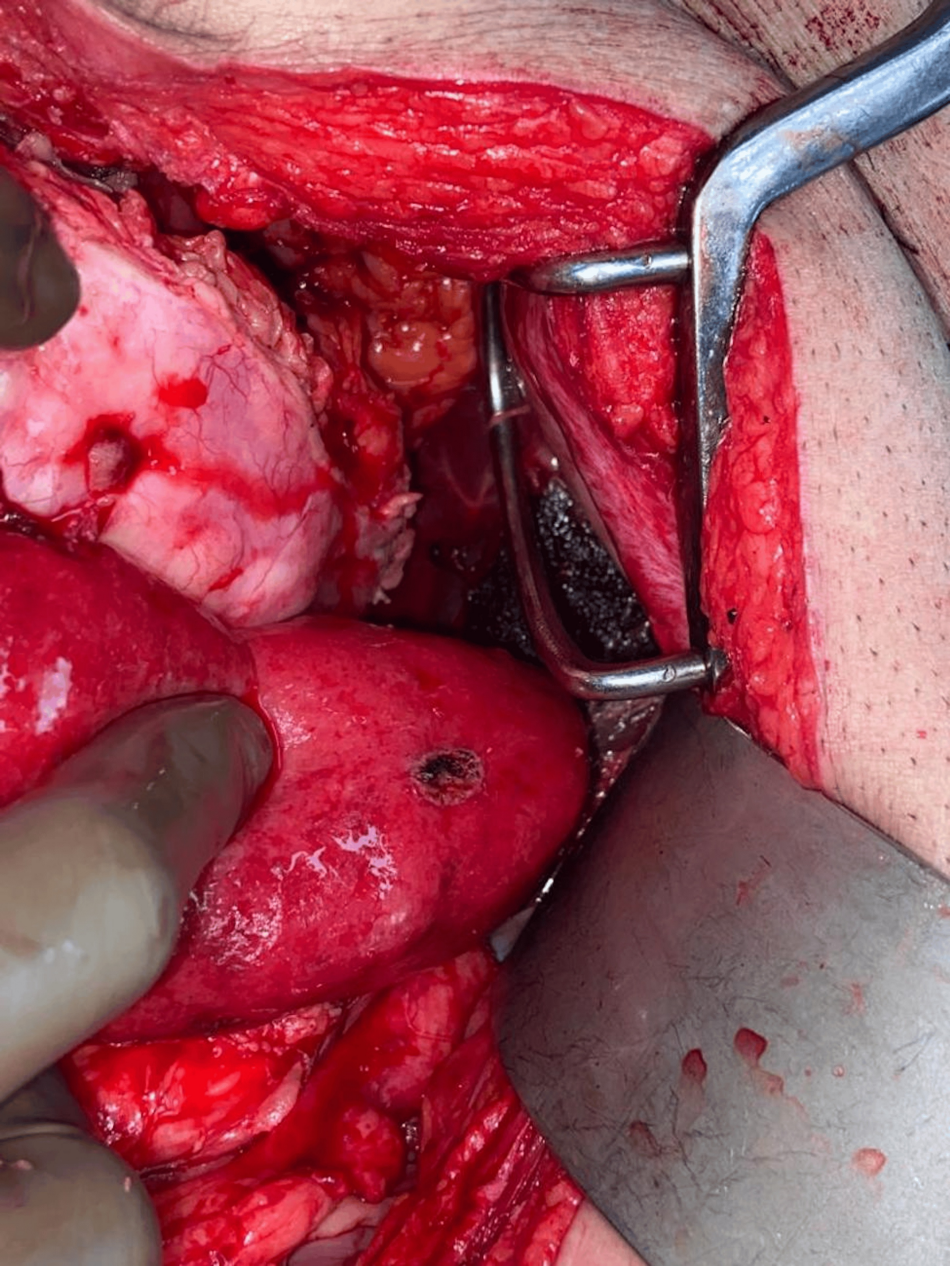
Intraoperatively after excision of mass

A 68-year-old male came with a two-year history of heaviness and swelling in the right flank, progressively increasing in size. The patient also reported intermittent constipation but had no other significant symptoms. Physical examination revealed a distended abdomen with a palpable, tense mass of unclear margins. Laboratory results were unremarkable. An abdomen pelvis CT scan revealed a 32x22x24 cm mass in the right abdominal cavity, primarily retroperitoneal, causing posterior displacement of the right kidney, medial displacement of the pancreas, anterior displacement of the right colon, and extension to the liver surface. MRI abdomen was done which confirmed the CT findings (Figure 1). No evidence of any metastasis was seen in the investigations done. A laparotomy was done for the patient, and a large mass from the right retroperitoneum was completely resected (Figure 2). 

**Figure 3:**
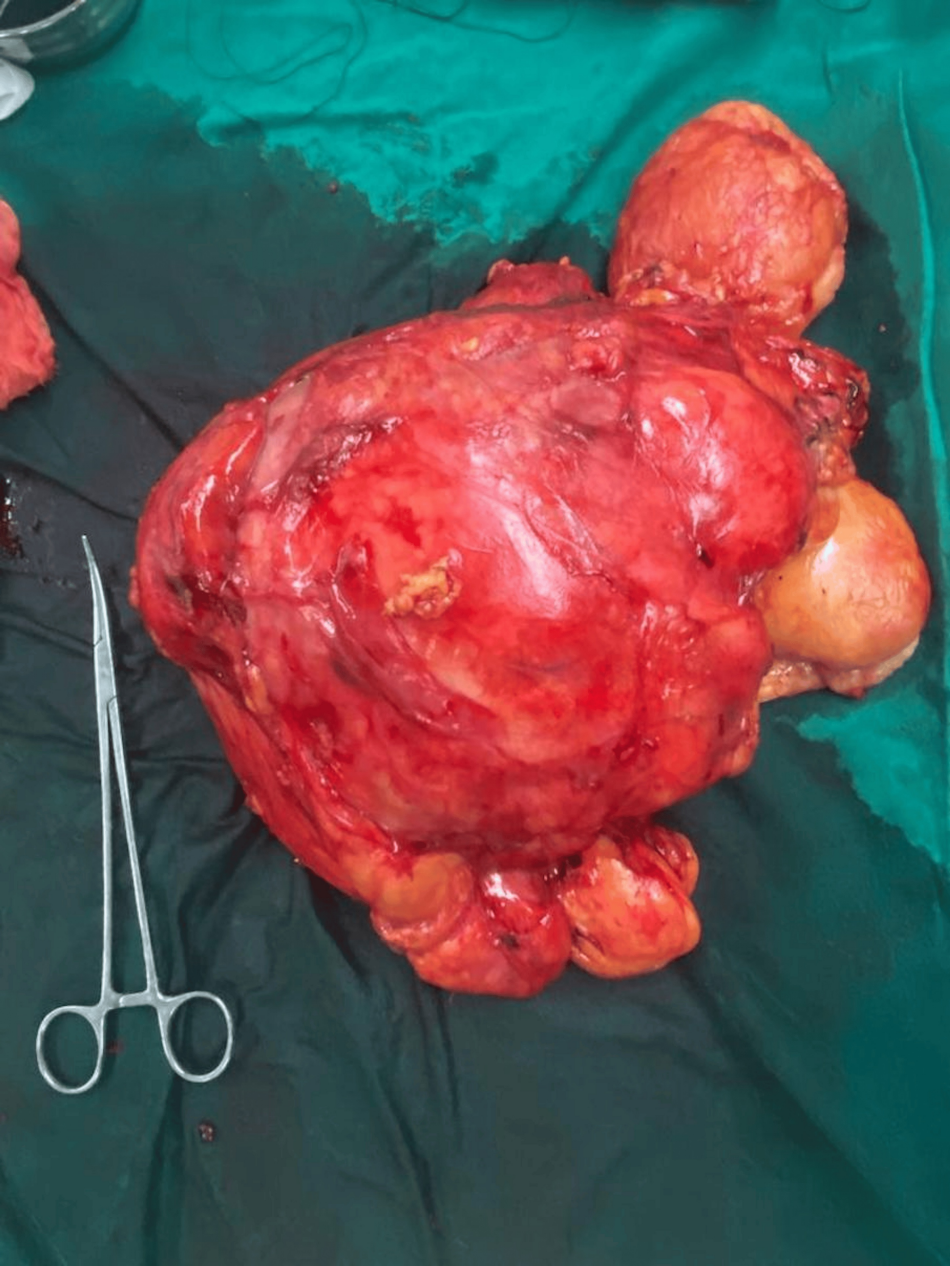
Excised mass in toto

The resected mass measured approximately 33x23 cm and weighed 22 kg (Figure 3).

Histopathological analysis confirmed a well-differentiated Grade I retroperitoneal tumor. The discharge of the patient was on the fourth day after surgery and remained free of recurrence or additional complaints during a 12-month follow-up.

## Discussion

Retroperitoneal liposarcomas typically occur in patients aged 40 to 60 years, with equal gender distribution [5]. These tumors can grow extensively within the retroperitoneal space without compressing vital organs due to the lack of bony boundaries. Clinical symptoms such as abdominal discomfort, weight loss, and abdominal lumps usually indicate advanced tumor growth and involvement of surrounding structures. Retroperitoneal liposarcomas are generally low to intermediate in malignancy, with hematogenous metastasis being rare at diagnosis. The lungs are the most common site for distant metastases. Liposarcomas are classified into four types: dedifferentiated, pleomorphic, well-differentiated, and myxoid/round cell. Dedifferentiated and pleomorphic types are highly malignant and aggressive, while well-differentiated and myxoid/round cell types are of low to moderate malignancy [6]. The well-differentiated subtype, as in this patient, is associated with a favorable prognosis and a five-year survival rate of 83% to 90% [4].

Complete surgical resection is the mainstay treatment for retroperitoneal liposarcoma, as the effectiveness of neoadjuvant or adjuvant chemotherapy and radiotherapy remains debatable [2,7]. Chemotherapy shows varying responses depending on the liposarcoma subtype, with well-differentiated liposarcoma generally exhibiting poor response. Radiotherapy has not demonstrated significant benefits in these cases. The goal of surgery is to achieve complete resection, including any invaded organs, to improve survival outcomes [8]. Achieving clear margins can be challenging, particularly in well-differentiated liposarcomas. Combined organ resection may be necessary, with the kidney and colon being the most commonly removed organs. Local recurrence is the primary cause of mortality, making complete resection and thorough follow-up crucial. Regular CT scans are recommended to monitor for recurrence.

## Conclusions

Giant retroperitoneal liposarcoma, although rare, must be considered in patients presenting with substantial abdominal masses, as early identification is critical for timely intervention. These tumors, especially when well-differentiated, require prompt and complete surgical resection to improve the chances of a favorable prognosis. The complexity of such cases is not limited to the surgery itself; consistent and long-term follow-up is equally crucial. Regular monitoring is essential for the early detection of any potential recurrences, which can often pose significant challenges in management due to the tumor's tendency to grow large before becoming symptomatic again.
